# Inferences about women’s traits influence the judgment of their eye contact

**DOI:** 10.3758/s13423-025-02704-7

**Published:** 2025-05-27

**Authors:** Manman Zhai, Jari K. Hietanen

**Affiliations:** Human Information Processing Laboratory, Faculty of Social Sciences/Psychology, Tampere University, 33014 Tampere, Finland

**Keywords:** Trait inference, The cone of direct gaze (CoDG), Social desirability, Self-referential positivity, Female face preference

## Abstract

We investigated whether trait inference about others’ social desirability influences perceivers’ judgments of eye contact with them, measured by the cone of direct gaze (CoDG) width—the range within which slight gaze deviations are perceived as direct. We also explored how the effect is modulated by participants’ gender and gazers’ sex. In Experiment 1, participants read descriptions of a likeable female and an unlikeable male (or vice versa). Subsequently, they judged whether they were being looked at or not by these individuals displaying various gaze directions. In Experiment 2, participants inferred trait desirability and performed eye contact tasks sequentially for two faces of the same sex, either beginning with a likeable face followed by an unlikeable face or vice versa. In Experiment 3, participants first performed eye-contact judgment for an individual without trait manipulation. They then inferred trait desirability for a second same-sex individual whose traits were manipulated as likeable or unlikeable, followed by eye-contact judgment for that individual. Results showed that CoDG width was wider for female faces associated with socially desirable traits than for those associated with socially undesirable traits. Desirable traits inferred from women’s social behaviors may align with individuals’ expectations of stereotypical women, enhancing their perceived likeability. The widened CoDG may result from individuals’ approach-related action tendency, self-referential positivity bias, or a combination of both mechanisms.

**Supplementary Information:**

The online version contains supplementary material available at 10.3758/s13423-025-02704-7.

## Introduction

In real-world interpersonal communication, eye gaze is a powerful social signal that serves various functions, such as expressing intimacy and regulating social interactions (Kleinke, 1986). In interaction, the perception of eye gaze can be influenced by many intraindividual and interindividual variables, including observers’ and gazers’ personal characteristics (e.g., age, gender, personality traits), and the relational dynamics between them (e.g., friendship, romantic relationship, dependency; Kleinke, 1986). In the present study, we investigate how inferred desirability of a gazer’s personality influences observers’ perception of eye contact. We also investigate, the potential roles of the observer’s and the gazer’s gender, aiming to clarify how the observer’s gender and their gender-related stereotypes about men and women shape their perception of direct gaze.

Direct gaze (i.e., a social partner’s gaze directed toward the observer) is a highly important cue in interactive situations. Another individual’s direct gaze conveys that the observer is within the gazer’s attentional focus. Being looked at elicits affective reactions in the observer. The valence of affective reactions depends on many contextual factors. One such factor is perceivers’ cultural background. For example, East Asians evaluate direct gaze displayed by physically present people as angrier, less approachable, and more unpleasant as compared with Western Europeans (Akechi et al., 2013; but see Uono & Hietanen, 2015, for no cultural differences when investigated with static images). Also, individuals with high social anxiety are more likely to perceive direct gaze as threatening (Wieser et al., 2009). Moreover, direct gaze has been shown to intensify perceivers’ negative affective reactions to angry facial expressions displayed by the gazers (Adams & Kleck, 2003, 2005; Sander et al., 2007). However, empirical research has shown that, in neutral laboratory conditions, direct gaze from individuals displaying neutral expressions elicits positively valenced affective reactions among general population (Chen, Helminen, et al., 2017; Lawson, 2015; for a review, see Hietanen, 2018), and activates the brain systems associated with approach motivation (Hietanen et al., 2008; Uusberg et al., 2015). The direct gaze also captures and holds the observer’s attention towards the gazer’s face, which may further facilitate socially relevant affective and cognitive functions, such as emotion categorization (Bindemann et al., 2008) and empathy (Breil & Böckler, 2021; McCrackin & Itier, 2021).

Despite the sensitivity to a direct gaze from early infancy (Farroni et al., 2002), humans are not perfectly accurate in determining whether they are being looked at or not. Actually, there is a range within which slight gaze deviations are accepted as eye contact, termed the cone of direct gaze (CoDG; Gamer & Hecht, 2007). A wide CoDG, if not taken to the extreme, is beneficial for both the observer and the gazer in establishing social connectedness. Through accepting a considerably large range of gaze deviations as direct gaze, an observer can avoid the cost of missing a mutual gaze and maximize the opportunities to interact with the gazer. Several factors influencing social interaction have been demonstrated to modulate CoDG width, such as characteristics related to the observer (e.g., social anxiety; Chen, Nummenmaam, et al., 2017; Harbort et al., 2013; Jun et al., 2013) as well as those related to the gazer. In interpersonal interactions, for example, a gazer’s face can convey important information influencing the judgment of gaze direction. Previous studies have shown that CoDG width is wider for attractive (vs. unattractive) faces (Kloth et al., 2011) and happy (vs. neutral) faces (Gianotti et al., 2018; Lobmaier & Perrett, 2011; Lobmaier et al., 2008). Interpreting the attention of an attractive or a happy person as being directed at oneself affords opportunities of social rewards, such as self-enhancement and elevated self-esteem (a self-positivity bias; Alicke & Sedikides, 2009).

Acquired information about other people’s personality traits can also be used to determine whether to interact with them or not. Individuals’ personality traits affect their social relationships (Asendorpf & Wilpers, 1998). Traits related to social desirability, such as friendliness or arrogance, can be spontaneously inferred through observing people’s behavior or reading verbal descriptions of their behavior (Uleman et al., 2008; Winter & Uleman, 1984). This process, termed spontaneous trait inference (STI), constitutes the basis of the impressions we form about others (Asch, 1946; Carlston & Skowronski, 2005; Heider, 1958; Shimizu, 2017). Such inferred traits can influence observers’ cognitive processes and behaviors (Crawford et al., 2013; Hastie & Kumar, 1979; Kobayashi et al., 2022; McCarthy & Skowronski, 2011; Snyder, 1984; White & Carlston, 1983; Wildman & Ramsey, 2022). For example, inferred desirable traits may serve as cues for observers to initiate approach-related behaviors, whereas inferred undesirable traits may signal that a person should be avoided, thereby minimizing the likelihood of a negative interpersonal encounter. The tendency to approach or avoid a person can be reflected in an observer's judgment about whether they are being looked at by the person. Inferred desirable traits that activate the observer’s approach tendency toward someone may increase the probability that the person is perceived as making eye contact. In contrast, inferred undesirable traits that activate the observer’s avoidance tendency may decrease this probability. Additionally, previous research on modulatory factors related to gazers has exclusively focused on facial information (e.g., facial attractiveness: Kloth et al., 2011; emotions: Gianotti et al., 2018; Lobmaier et al., 2008; Lobmaier & Perrett, 2011), which is visible to observers. However, it remains unclear whether and how nonfacial information, such as prior trait knowledge about a gazer formed through trait inference, influences an observer’s CoDG when the gazer displays a neutral expression, devoid of contextual cues that imply trait desirability. Therefore, in the current study, we aimed to investigate whether and how the perception of CoDG is influenced by the gazer’s trait desirability. In addition, we were also interested in studying how this effect is modulated by the observer’s gender and the gazer’s sex.

Previous research has shown gender differences in perceiving eye contact. Compared with women, men are more prone to judge others making eye contact with them and show a wider CoDG (Hietanen et al., 2022; Huang et al., 2022; Lobmaier & Perrett, 2011; Lobmaier et al., 2008; Zhai et al., 2025). Moreover, participants’ gender also modulates the effects of contextual factors (e.g., facial expression) on the perception of ambiguous gaze direction (Huang et al., 2022; Shi et al., 2020; Zheng et al., 2021). For instance, men have a wider CoDG for angry faces compared with fearful faces and neutral faces, whereas women’s CoDG width for both angry and fearful faces is significantly wider than for neutral faces, but the CoDG width between fearful faces and angry faces does not differ (Huang et al., 2022). Such emotional effects of facial expressions on CoDG width, qualified by participant gender, may arise from gender differences in the strategies used to evaluate and cope with threat (Matud, 2004). Men may distinguish between different sources of threats and use different coping strategies accordingly. It is possible that men associate an angry face with an interpersonal conflict, and this leads to widened CoDG because of attempts for adaptive coping. In contrast, in a dangerous context indicated by a partner’s fearful expression, men may not necessarily rely on others’ gaze direction. Compared with men, women are more sensitive to threats (Lambert et al., 2016; Robinson et al., 2021) and show greater self-protective responses (Huntsinger et al., 2022) regardless of whether the threat stems from interpersonal interactions or from external environment. Consequently, women may adaptively evaluate both facial anger and fear as directed to the self. Even though STI effects are robust regardless of participants’ gender (Bott et al., 2024), there may also be gender differences in how people use information about other people’s personality traits in the perception of their facial cues. Specifically, women may use acquired information about others to estimate the possibility of relationship formation and maintenance, whereas men may use this information to enhance their own uniqueness through social comparisons (Cross & Madson, 1997).

It is also important to investigate the role of face sex in gaze cone studies, a topic that has often been overlooked. Previous studies focusing exclusively on male faces, whether virtual or real (Ewbank et al., 2009; Gamer & Hecht, 2007; Gamer et al., 2011; Harbort et al., 2013; Jun et al., 2013), were unable to explore the effects of face sex. Although more recent studies have included both male and female faces (Chen, Nummenmaa, et al., 2017; Lyyra et al., 2017; Syrjämäki et al., 2020; Uono & Hietanen, 2015; Wastler & Lenzenweger, 2018), the impact of face sex has not been incorporated into data analyses. As opposed to male faces, female faces are generally associated with positive social and psychological traits, such as warmth (Delacollette et al., 2013), trustworthiness (Wirth & Wentura, 2023), and concern for others (Williams & Best, 1982). Thus, people may prefer female faces because of the associated social and psychological benefits with women. Supporting this notion, research has found that both men and women under low social support conditions show stronger preferences for feminine faces, as femininity is perceived as an indication of high-quality social support (Watkins et al., 2012). Given the influence of preferences for female faces on social perception, our study included the stimulus face sex as a key variable. We aimed to investigate whether the gazer’s sex would modulate the influence of inferred traits on the perception of direct gaze.

In the present research, we examined whether trait inference regarding others’ social desirability influences the perception of direct gaze, as measured by the CoDG width. Additionally, we investigated whether participant’s gender and the stimulus face sex modulate the effect of inferred trait desirability on direct gaze perception. To address these questions, we used an impression-formation task in which participants read verbal descriptions of individuals to infer their trait desirability. Direct gaze perception, indexed by CoDG width, was measured through an eye-contact judgment task. Participants viewed a series of face images of the described individuals, each with varying gaze deviations, and judged whether they were being looked at or not. In Experiment 1, during the impression-formation task, participants were presented with a female described as likeable and a male described as unlikeable (or vice versa). Subsequently, in the eye-contact judgment task, the participants were asked to judge whether they were being looked at or not by these two individuals. In Experiment 2, the two stimulus faces were of the same sex. The impression-formation task and the subsequent eye-contact judgment task were presented separately for these two (likeable and unlikeable) persons. Experiment 3 also used same-sex stimulus faces. Also in this experiment, the faces of the two persons were presented separately to the participants, but no trait information was provided for the first presented person, and the participants completed the eye-contact judgment task for this individual (trait-neutral identity) without any trait manipulation. Subsequently, participants were presented with a second identity described as either likeable or unlikeable in the impression-formation task, followed by the eye-contact judgment task. Across all three experiments, the results provided evidence that inferred traits associated with female faces had an effect on participants’ eye-contact perception; CoDG width was wider for female faces associated with socially desirable traits compared with those associated with socially undesirable traits.

## Experiment 1

In Experiment 1, to decrease the cognitive load of memorizing the association between the faces of the two persons and the descriptions of their social desirability, for each participant, one of the faces was a female face and the other was a male face. The Desirability × Sex of the Face matching was counterbalanced across the participants. We hypothesized that participants’ CoDG width would be wider for a person previously described with socially desirable behaviors compared with a person described with socially undesirable behaviors. Replicating previous findings, men were expected to have a wider CoDG than women, but we did not form a definitive hypothesis regarding whether participants’ gender would modulate the effects of inferred traits on CoDG width. Finally, we also tested (without a hypothesis) whether the Desirability × Sex of the Face (desirability–face sex mapping) had an influence on the results.

### Methods

#### Participants

We recruited 382 participants (average age: 39 years; range: 18–73 years) online via Prolific (https://www.prolific.co/) (Palan & Schitter, 2018). Only participants who reported their gender as man or woman received the invitation to this study. In a previous study, detecting the gender difference in CoDG width with an effect size of *d* = 0.38 (Hietanen et al., 2022) required a sample size of 220 participants (110 for each gender) to achieve a statistical power of 0.80 at an alpha level of 0.05. As online data collection may result in significant data loss, we decided to recruit a larger sample. Using the effect size of the interaction between participants’ gender and the desirability of inferred traits (*η*_*p*_^*2*^ = 0.02), we also conducted a post hoc power analysis with G*Power (Faul et al., 2007). The result indicated that with the final sample (*N* = 350; 173 women: *M*_Age_ = 38.38 years, *SD* = 14.00; 177 men: *M*_Age_ = 39.24 years, *SD* = 14.03; the age difference between women and men was not significant: *t*(348) = − 0.57, *p* = 0.57), we achieved a power of 98% with an alpha level of 0.05 to detect the two-way interaction. Participants were included if a) they lived in the United Kingdom, b) their first language was English, and c) their Prolific approval rate was 100%. Additionally, participants were required to have a normal or corrected-to-normal vision and no neurological or psychiatric disorders. The link to the present study was not accessible to participants who used tablets or smartphones. The principles outlined in the Declaration of Helsinki were followed when conducting this research. The Ethics Committee of Tampere region approved that the present study was exempt from a formal ethical review and evaluated that the present study followed good scientific practice before the submission, and a statement regarding their decision was obtained (Date: 04/06/2024).

#### Stimuli and programming of the experiment

**Trait-implied words and sentences.** Ten trait adjectives (five words describing traits rated socially desirable: caring, friendly, generous, helpful, and reliable; five words describing traits rated socially undesirable: arrogant, dishonest, hateful, rude, and violent) were selected from an English list of traits (ELoT; Britz et al., 2022). The selection of the words was based on the normative data of valence/desirability/observability ratings reported in this database (see Table S1 in Supplemental File). Following the selection of the words, four to five sentences describing the trait-implied behaviors were created for each word using Microsoft Bing Chat Enterprise AI chatbot. The authors of the present study discussed and selected together one sentence for each word to be used in the experiment. The sentences that were finally selected did not contain the words used to generate them (e.g., caring, “She/He always listens attentively to others’ feelings and problems.”). See all the sentences in Supplemental File (Table S2).

**Fig. 1:**
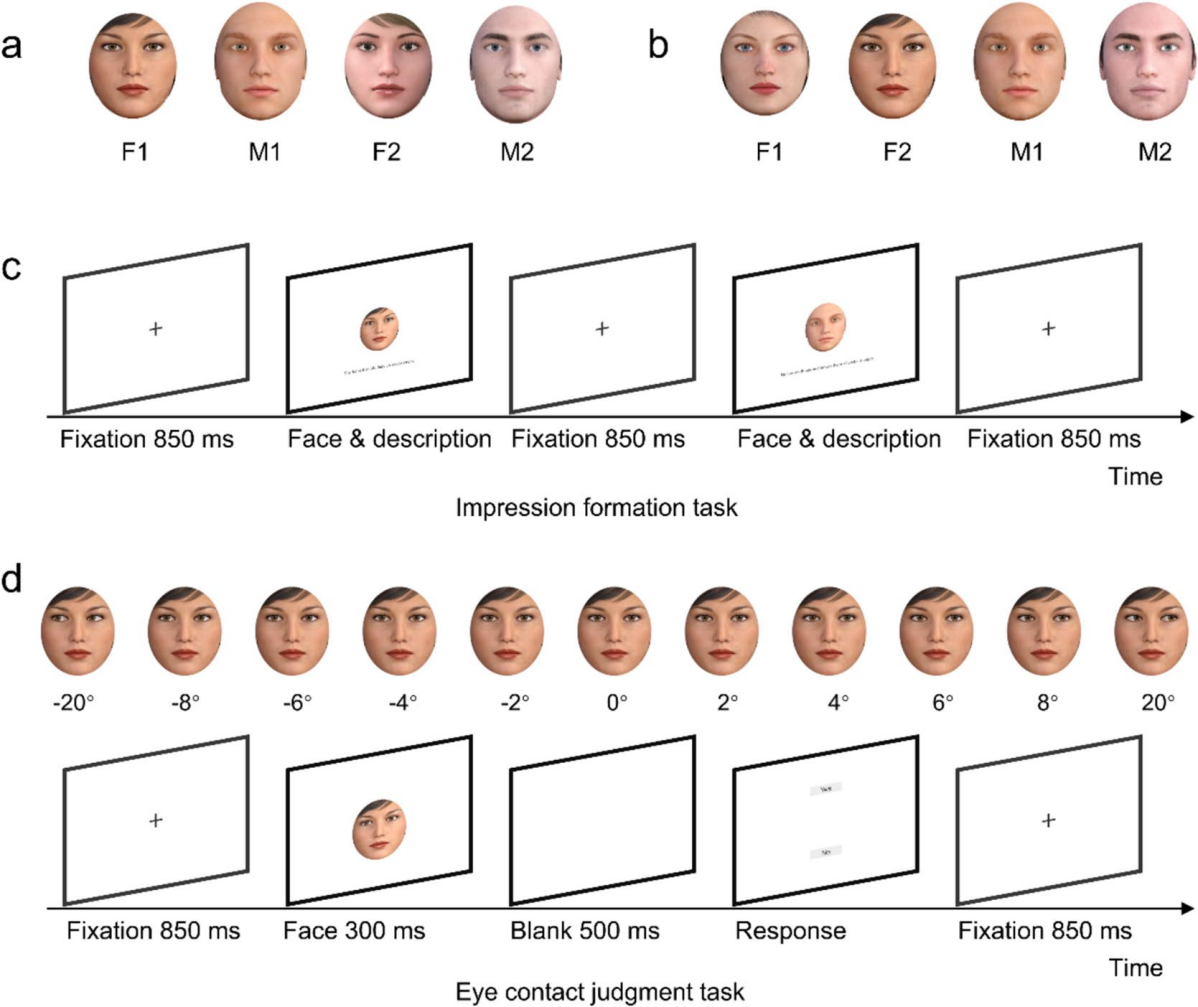
Face stimuli in Experiment 1 (**a**), face stimuli in Experiments 2 and 3 (**b**), a schematic flow of the events on trials in the impression-formation task (**c**) and in the eye-contact judgment task (**d**)

**Face stimuli.** The face stimuli were four neutral faces (two females: F1/F2; two males: M1/M2), which were created using 3D animation software (Digital Art Zone 3D studio, Daz 3D) (https://www.daz3d.com/). The gaze directions varied at a step of 2˚ from direct gaze (0˚) to leftward and rightward averted gaze (2˚, 4˚, 6˚, 8˚). For attention check purpose, gaze stimuli of 20˚ were also included. Mirror images of the face stimuli were created by flipping the original images horizontally to avoid the potential influence of facial asymmetry. An oval area of the face region was cropped to eliminate the possible influence of hair and ears (see Fig. 1a). Each face model was evaluated as equally attractive by women and men in another stimulus assessment study (see Table S3 in Supplemental File for the attractiveness ratings and corresponding statistics). Four versions of experimental tasks were created. The combinations of face stimuli for the four versions were: F1 combined with desirable traits and M1 combined with undesirable traits (Version 1; + F‒M); F1 combined with undesirable traits and M1 combined with desirable traits (Version 2; + M‒F); F2 combined with desirable traits and M2 combined with undesirable traits (Version 3; + F‒M); F2 combined with undesirable traits and M2 combined with desirable traits (Version 4; + M‒F). Given that each face was shown only for a brief duration (300 ms) in the eye-contact judgment task, we wanted to make sure that the participants were able to differentiate the facial identities (and traits) of the stimulus faces. Therefore, in each version, we presented participants with the faces of two different persons only. Furthermore, since both the two female and two male faces were created from the same templates in Daz 3D studio, we also decided not to show faces of the same sex for the participants in Experiment 1, as it might have been difficult to differentiate between two identities of the same sex.

Lab.js (https://lab.js.org/), a free, open, and online study builder, was used to program the experimental tasks (Henninger et al., 2022). The study was uploaded to Open Lab (https://open-lab.online/) (Shevchenko, 2022) to generate an external link which was inserted on Prolific, the platform used to recruit the participants.

#### Experimental procedure

People registering as participants on the Prolific platform received the invitation message, which contained the aims and duration of the study, a brief description of what they were required to do in the study, and the abovementioned prerequisites for participation. The participants were told that clicking the external link to start the study meant that they gave informed consent to participate in the study and that the results and anonymized data can be published. After entering the study, they completed the tasks in a fixed sequence: impression-formation task, trait-judgment task, eye-contact judgment task, and likeability rating task. Before each task, detailed information was presented to guide the participants on how to perform the tasks.

**Impression-formation task.** In this task, the faces of two persons with verbal descriptions (sentences) of their social behaviors were randomly presented. Participants were required to passively read the sentences describing these two people. There were five sentences for each person. Each trial started with a fixation point in the center of the screen (850 ms), which was followed by a person’s face and a sentence below it, both presented simultaneously for 5 s. After this, the next trial started (see Fig. 1c).

**Trait-judgment** **task**. After completing the impression-formation task, participants were presented with the two faces they saw previously, along with the ten trait words that were used to generate the verbal descriptions of social behaviors in the previous task (note that these words did not appear in the descriptions of behavior). All the words were displayed below each face on the screen. Participants were required to evaluate the extent to which the words (listed in alphabetical order) described the personalities of the two people. The evaluation was made on a 1–7 scale; 1 represented “does not describe the person at all” and 7 represented “describes the person extremely well.” The aim of this task was to reinforce the impressions formed about the two persons.

**Eye-contact judgment task.** First, the participants were required to adjust the distance between them and the screen at full arm’s length and ensure they were seated directly in front of the computer screen. After that, they started the eye-contact judgment task. The task consisted of two blocks, between which participants could have a self-paced break. In each block, the participants were presented with the same female and male identities they saw in the impression-formation task. Each face was presented with the averted gaze directions (left and right: 2˚, 4˚, 6˚, and 8˚) four times and the direct gaze (0˚) eight times, with half in the original versions and another half as mirror images, thus resulting in 80 trials. Additionally, we included faces with the gaze direction of 20˚ (left and right) to serve as attention check trials. These stimuli (including mirror images) were presented once for each identity, resulting in eight trials. Thus, in total, there were 88 trials in each block. Each trial started with an 850-ms fixation followed by a picture of face displaying an averted gaze or direct gaze for 300 ms. Subsequently, a blank screen was presented for 500 ms, followed by a response screen waiting for participants’ judgment of whether “the person in the picture is or is not looking directly at you.” On the response screen, the word “yes” appeared in the upper part of the screen, while the word “no” appeared in the lower part of the screen. Correspondingly, participants pressed “y” on their keyboards if they felt that the person was looking at them and they pressed “n” if they felt that the person was not looking at them (see Fig. 1d).

**Likeability rating task and personal information.** Participants were required to rate the likeability of the two persons they saw in the tasks on a scale of 1–7. 1 represented “extremely unlikeable” and 7 represented “extremely likeable.” The aim of this task was to check whether the impression formed about the two persons lasted for about 10 min. Finally, participants were asked to report their gender, age, whether they had participated in an eye-contact judgment study before, and whether they experienced any technical issues that might have influenced their results. Upon the completion of this final report, participants were thoroughly debriefed and informed about the aims of all the tasks.

#### Data analysis

Complete data from 382 participants (184 women and 198 men) were collected. Before the calculation of CoDG width for every participant, the 20˚ gaze trials were removed, and participants were excluded if the percentage of “yes” responses to the gaze direction of 8˚ (leftward and rightward averted gaze) was more than 50%, or the percentage of “yes” responses to direct gaze (0˚) was less than 50%. Based on the criteria, eight participants were excluded. Another two participants reporting technical issues were also excluded.

The data of the eye-contact judgment task were analyzed by calculating a point of subjective equality (PSE; the point at which the gaze direction has a 50% probability to be judged as a direct gaze or averted gaze) for each participant. CoDG width was approximated as the PSE multiplied by two (to cover both the left and right sides). The PSE was calculated using a binary logistic regression model for each participant. The calculation was conducted in R using the *glm* function and *logit* link function, in which the gaze direction (0˚/2˚/4˚/6˚/8˚) was the independent variable, and the dichotomous responses to whether being looked at or not were the dependent variable. Following the formula below, the PSE equals − *β*_0_/*β*_1_ with a *p* value (the probability of a “yes” response) of 50%.

1 $$\text{log}\left(\frac{P}{1-P}\right)= {\beta }_{0}+ {\beta }_{1}x.$$

After the calculation of CoDG width, the goodness of model fit for the binary logistic regression models was assessed by examining the deviance residuals. A deviance residual higher than the corresponding degree of freedom indicates that the model is a poor fit for the data. Based on this criterion, another 21 participants were excluded. Based on visual inspection, one participant was excluded due to the negative value of CoDG. Finally, two more participants were excluded as outliers, as their CoDG width was not within three standard deviations from the mean. In sum, the final data analysis from the eye-contact judgment task were based on the data from 350 participants (Ethnicity (White/Black/Asian/Mixed/Other): Men: 150/3/16/5/3; Women: 151/4/9/7/2). We also checked participants’ responses on attention check trials (16 trials of 20˚ gaze direction), and the average error rate on the catch trials was 0.45%. One participant made three erroneous responses, one participant made two, 20 participants made one, and the remaining 327 participants made no erroneous responses. The analysis of the likeability ratings was based on participant’s ratings of the likeable person and unlikeable person on a single likeability (1–7) scale.

To examine whether associating desirable versus undesirable traits with a female versus male face influenced the results, a third variable of desirability–face sex mapping was included as a between-subjects factor. Mixed design of 2 [Desirability of inferred traits: desirable vs. undesirable] × 2 [Participant gender: woman (*n* = 173) vs. man (*n* = 177)] × 2 [Desirability–face sex mapping: likeable female face–unlikeable male face: + F‒M (*n* = 179) vs. likeable male face–unlikeable female face: + M‒F (*n* = 171)] analyses of variance (ANOVAs) were performed on the gaze-cone data and the likeability-rating data. As the age distribution of the sample recruited online exhibited a wide age range, and as previous studies have shown a positive association between age and individual CoDG width (Hietanen et al., 2022), we included age as a covariate in the analysis of the gaze-cone data. The effect of participants’ age as a covariate was significant [*F*_(1,345)_ = 18.29, *p* < 0.001, *η*_*p*_^*2*^ = 0.05], showing that the older the participant, the wider the CoDG.^1^

As mentioned above, the trait-judgment task was aimed to reinforce impressions of individuals whose trait desirability was manipulated. Although the effectiveness of manipulation could have been confirmed using data from this task, it would have remained uncertain whether the effect of manipulation would persist through the subsequent eye-contact judgment task. Therefore, the effectiveness of the trait desirability manipulation was evaluated based on participants’ likeability ratings collected at the end of the experiment. Data from the trait-judgment task were analyzed as supplementary information, with the results reported and discussed in the Supplemental File (Supplemental Analysis 1, 2, and 3 for trait-judgment tasks in Experiments 1, 2, and 3). When reporting the results from the likeability rating task in the main text, we focused on the main effect of trait desirability and its interactions with other variables as well as the main effect of face sex. Complete ANOVA results are provided in the Supplemental File (Table S5).

### Results

#### Likeability

A significant main effect of desirability [*F*_(1,346)_ = 196.48, *p* < 0.001, *η*_*p*_^*2*^ = 0.36] indicated that participants evaluated the persons previously associated with desirable traits [*M* = 5.18, *SE* = 0.06] more likeable than the persons previously associated with undesirable traits [*M* = 3.80, *SE* = 0.07]. There was also a significant interaction between desirability and desirability–face sex mapping [*F*_(1,346)_ = 39.42, *p* < 0.001, *η*_*p*_^*2*^ = 0.1]. The effect of desirability was significant for both pairs [*p* values < 0.001], but it was greater for the + F‒M pairs [*M*_+F_ = 5.47, *SE* = 0.08; *M*_‒M_ = 3.47, *SE* = 0.10] than the + M‒F pairs [*M*_+M_ = 4.89, *SE* = 0.09; *M*_‒F_ = 4.13, *SE* = 0.10]. Further analyses showed that likeable female faces in + F‒M pairs were rated as more likeable than likeable male faces in + M‒F pairs [*p* < 0.001], while unlikeable female faces in + M‒F pairs were rated as more likeable than unlikeable male faces in + F‒M pairs [*p* < 0.001].

#### The cone of direct gaze (CoDG)

**Fig. 2:**
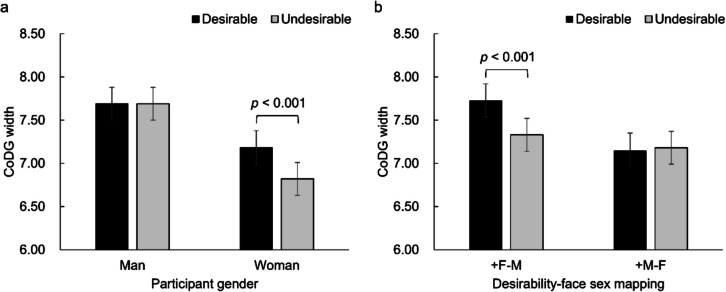
Means and standard errors of CoDG width as a function of desirability of traits and participant gender (**a**) and desirability of traits and desirability–face sex mapping (**b**)

Table 1 shows the means (*SD*s) of CoDG width in each cell of Desirability of inferred traits × Participant gender × Desirability–face sex mapping. The results showed a significant main effect of desirability [*F*_(1,345)_ = 4.15, *p* = 0.042, *η*_*p*_^*2*^ = 0.01], indicating that the CoDG width was wider for likeable people [*M* = 7.44, *SE* = 0.14] as compared with unlikeable people [*M* = 7.26, *SE* = 0.13]. A significant main effect of participant gender [*F*_(1,345)_ = 6.51, *p* = 0.011, *η*_*p*_^*2*^ = 0.02] showed that men [*M* = 7.67, *SE* = 0.18] had a wider gaze cone than women [*M* = 7.02, *SE* = 0.18]. Furthermore, a significant interaction between desirability and participant gender [*F*_(1,345)_ = 6.03,* p* = 0.015, *η*_*p*_^*2*^ = 0.02] revealed that the CoDG width was significantly wider for seeing likeable people [*M* = 7.20, *SE* = 0.19] versus unlikeable people [*M* = 6.84, *SE* = 0.19; *p* < 0.001] in women, whereas in men, there was no significant difference in CoDG width between likeable people [*M* = 7.68, *SE* = 0.19] and unlikeable people [*M* = 7.67, *SE* = 0.19; *p* = 0.981] (Fig. 2a). Another significant interaction between desirability and desirability–face sex mapping [*F*_(1,345)_ = 9.31,* p* = 0.002, *η*_*p*_^*2*^ = 0.03] revealed that participants’ CoDG width was wider for likeable female faces [*M* = 7.71, *SE* = 0.19] than for unlikeable male faces [*M* = 7.32, *SE* = 0.19; *p* < 0.001] in + F‒M pairs, while there was no significant difference in CoDG width for likeable male faces [*M* = 7.16, *SE* = 0.19] and unlikeable female faces [*M* = 7.20, *SE* = 0.19; *p* = 0.694] in + M‒F pairs (Fig. 2b). The main effect of desirability–face sex mapping and the other interactions were not significant [all *p* value*s* > 0.05].

**Table 1:** Means (and standard deviations) of CoDG width by participant gender, desirability–face sex mapping, and desirability

Participant gender	Desirability–face sex mapping	Desirable mean (*SD*)	Undesirable mean (*SD*)
Man	+ F–M	7.94 (2.54)	7.74 (2.57)
	+ M–F	7.45 (2.56)	7.64 (2.43)
Woman	+ F–M	7.52 (2.75)	6.92 (2.87)
	+ M–F	6.84 (2.38)	6.73 (2.20)

### Discussion

Supporting the main hypothesis, the results revealed that participants’ CoDG width was wider when seeing a likeable person compared with an unlikeable person. The results also showed that men had a wider CoDG than women, replicating previous research (Hietanen et al., 2022; Huang et al., 2022). More interestingly, participants’ gender influenced the effect of desirability on CoDG width. Among men, CoDG width did not differ significantly when seeing a likeable person versus an unlikeable person, whereas women exhibited wider CoDG width when seeing a likeable person compared with an unlikeable person.

Somewhat unexpectedly, the results showed that the effect of inferred trait desirability on CoDG width was influenced by desirability–face sex mapping. The widening effect of desirable traits versus undesirable traits was observed in the + F‒M pairs, not in the + M‒F pairs. This suggests that the effect of trait desirability on CoDG width might be confounded by the stimulus face sex—possibly related to a preference for female faces. Such a preference was evident in the likeability rating task, where participants evaluated female faces as more likeable than male faces, regardless of whether the faces had been described with socially desirable or undesirable behaviors. Therefore, the preference for female faces likely contributed to the broader CoDG width observed in the eye-contact judgment task. This finding indicates that the broadened CoDG width cannot be exclusively attributed to the effect of trait desirability alone.

## Experiment 2

In the Method section of Experiment 1, we explained the reasons why we decided to use pairs of a female and a male face. However, as described above, this decision led to findings suggesting that the results of Experiment 1 did not convincingly indicate the effect of inferred trait desirability on the perception of direct gaze. To address this issue, in Experiment 2, we eliminated the confounding effect of stimulus face sex and presented faces of two same-sex persons, one associated with desirable traits and the other with undesirable traits. To minimize the possibility of direct comparison of the faces and the possible effect of physical facial features on CoDG width, the two faces were presented in separate blocks. Finally, we made some improvements to the facial images and verbal descriptions of the traits (see below).

### Methods

#### Participants

We recruited 352 participants for this experiment online via Prolific. The inclusion criteria were identical to those used in Experiment 1. In data analysis, exclusions were applied as follows: 18 participants gave “yes” responses to averted gaze at 8˚exceeding 50% or “yes” responses to direct gaze below 50%; 37 participants were excluded due to poor model fit; one participant reported technical issues; two participants had abnormal CoDG width values (less than 1˚ or negative values); and three participants had CoDG values outside the range of mean ± 3 standard deviations. After these exclusions, the final sample consisted of 291 participants (148 women: *M*_Age_ = 36.28 years, *SD* = 12.31; 143 men: *M*_Age_ = 37.51 years, *SD* = 13.55; Ethnicity (White/Black/Asian/Mixed/Other): Men: 113/7/10/10/3; Women: 120/12/10/2/4). The age difference between women and men was not significant (*t*(289) = − 0.81, *p* = 0.417). Finally, we also checked participants’ responses on attention check trials (16 trials of 20˚ gaze direction), and the average error rate on the catch trials was 0.32%. Two participants made two erroneous responses, 11 participants made one, and the remaining 278 participants made no erroneous responses.

#### Stimuli and procedure

This experiment consisted of four task versions for each face sex: (1) F1/M1 with desirable traits (Block 1) and F2/M2 with undesirable traits (Block 2) (+ 1‒2); (2) F2/M2 with undesirable traits (Block 1) and F1/M1 with desirable traits (Block 2) (‒1 + 2); (3) F1/M1 with undesirable traits (Block 1) and F2/M2 with desirable traits (Block 2) (‒1 + 2); (4) F2/M2 with desirable traits (Block 1) and F1/M1 with undesirable traits (Block 2) (+ 1‒2). Thus, in this experiment, target identity sex was manipulated as a between-subjects variable. Additionally, one female face and one male face were replaced with new faces to balance iris/pupil contrast across identities (see Fig. 1b). We also improved the descriptions of the target persons’ social behaviors by creating specific contexts in which participants could imagine themselves engaging in interpersonal interactions with the target person (e.g., “Imagine you were at a party where you knew no one. She greeted you with a warm smile and invited you to join her. She introduced you to others, making you feel welcome and included”; see Table S6 in Supplemental File for all vignettes). We reasoned that such self-relevant vignettes would be more effective in the context of dyadic interpersonal interactions and would enable participants to form particularly strong impressions of the people.

In this experiment, participants completed an impression-formation task, a trait-judgment task, and an eye-contact judgment task, in this order, separately for each identity. Half of the participants began with a likeable identity followed by an unlikeable identity, while the other half began with an unlikeable identity followed by a likeable identity. In the impression-formation task, on each trial, a face image and a short vignette below it describing the target person’s social behaviors were presented for 15 s or until terminated by the participant. All other aspects of the tasks were identical to those in Experiment 1. Finally, participants completed likeability ratings for the two identities.

#### Data analysis

The procedures for data cleaning and calculation of CoDG width were identical to those in Experiment 1. To examine whether the presentation order of associating desirable versus undesirable traits with an identity (first vs. second) influenced the results, a fourth variable, desirability–block order mapping, was included as a between-subjects factor. A mixed-design ANOVA was conducted with the following factors: 2 [Desirability of inferred traits: desirable vs. undesirable] × 2 [Participant gender: woman vs. man] × 2 [Face sex: female vs. male] × 2 [Desirability–block order mapping: likeable face first–unlikeable face second (+ 1‒2) vs. unlikeable face first–likeable face second (‒1 + 2)]. These analyses were performed on the gaze cone data and the likeability data. Additionally, age was included as a covariate in the analysis of the gaze cone data. The effect of participants’ age as a covariate was significant [*F*_(1,282)_ = 15.79, *p* < 0.001, *η*_*p*_^*2*^ = 0.05], showing that the older the participant, the wider the CoDG was.^2^ Complete ANOVA results for trait-judgment and likeability ratings are provided in the Supplemental File (Table S8).

### Results

#### Likeability

A significant main effect of desirability [*F*_(1,283)_ = 381.62, *p* < 0.001, *η*_*p*_^*2*^ = 0.57] indicated that participants evaluated the persons previously associated with desirable traits [*M* = 5.65, *SE* = 0.07] more likeable than those associated with undesirable traits [*M* = 2.90, *SE* = 0.09]. A significant main effect of face sex [*F*_(1,283)_ = 20.62, *p* < 0.001, *η*_*p*_^*2*^ = 0.07] showed that female faces [*M* = 4.48, *SE* = 0.06] received higher likability ratings than male faces [*M* = 4.07, *SE* = 0.07].

#### The cone of direct gaze (CoDG)

**Fig. 3:**
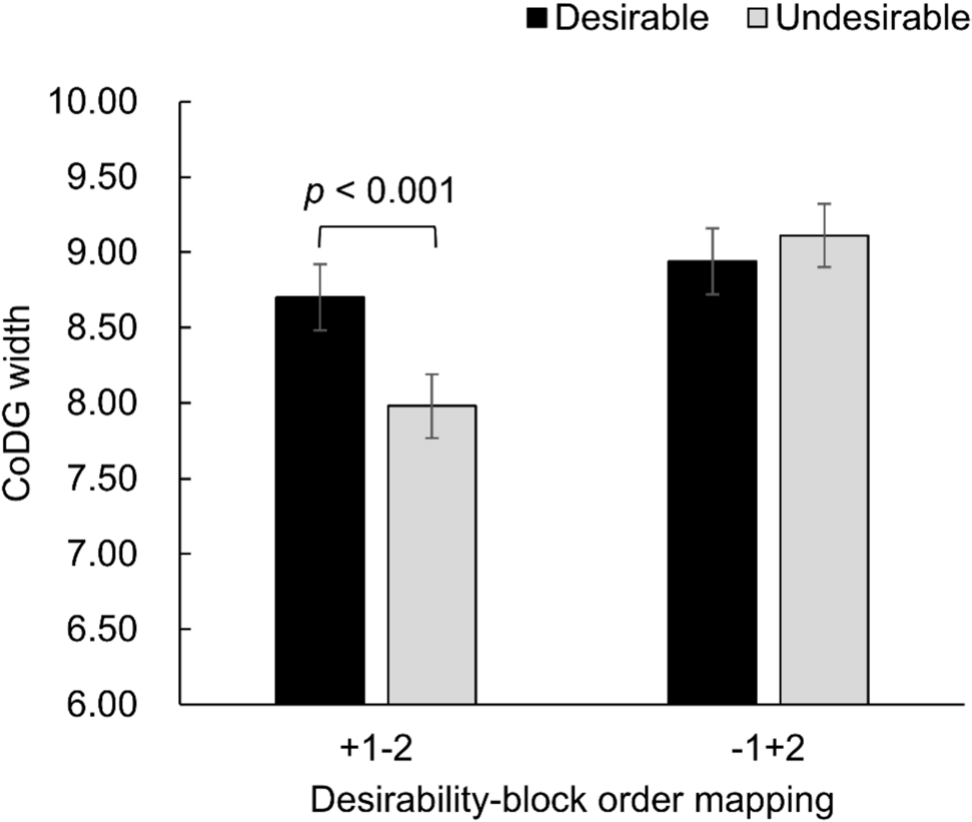
Means and standard errors of CoDG width as a function of desirability and desirability–block order mapping

Table 2 presents the means (*SD*s) of CoDG width in each cell of Desirability of inferred traits × Participant gender × Face sex × Desirability–block order mapping. The results showed that the main effect of desirability was not significant [*F*_(1,282)_ = 0.44, *p* = 0.507]. The main effect of participant gender was marginally significant [*F*_(1,282)_ = 3.83, *p* = 0.051, *η*_*p*_^*2*^ = 0.01], with men [*M* = 8.96, *SE* = 0.20] showing wider CoDG width than women [*M* = 8.41, *SE* = 0.20]. Additionally, a significant main effect of desirability–block order mapping [*F*_(1,282)_ = 6.35, *p* = 0.012, *η*_*p*_^*2*^ = 0.02] indicated that CoDG width was wider in ‒1 + 2 condition [*M* = 9.03, *SE* = 0.20] compared with the + 1‒2 condition [*M* = 8.33, *SE* = 0.20]. Most notably, desirability-block order mapping qualified the effects of desirability [*F*_(1,282)_ = 19.91, *p* < 0.001, *η*_*p*_^*2*^ = 0.07], participant gender [*F*_(1,282)_ = 4.36, *p* = 0.038, *η*_*p*_^*2*^ = 0.02], and face sex [*F*_(1,282)_ = 4.54, *p* = 0.034, *η*_*p*_^*2*^ = 0.02]. For the effect of desirability, the interaction demonstrated that in + 1‒2 condition CoDG width was significantly wider for likeable people [*M* = 8.69, *SE* = 0.21] compared with unlikeable people [*M* = 7.97, *SE* = 0.21; *p* < 0.001], whereas in ‒1 + 2 condition, CoDG width did not differ between likeable [*M* = 8.95, *SE* = 0.21] and unlikeable people [*M* = 9.12, *SE* = 0.21] (see Fig. 3). Other significant interactions are presented in detail in Supplemental File (Table S9).

**Table 2:** Means (and standard deviations) of CoDG width by participant gender, desirability–block order mapping, face sex, and desirability

Participant gender	Desirability–block order mapping	Face sex	Desirable mean (*SD*)	Undesirable mean (*SD*)
Man	+ 1 − 2	Female	8.75 (2.50)	8.27 (2.06)
		Male	9.91 (2.77)	8.73 (2.48)
	− 1 + 2	Female	9.13 (2.75)	9.24 (2.39)
		Male	8.79 (2.41)	9.07 (2.93)
Woman	+ 1 − 2	Female	7.46 (1.99)	6.63 (2.12)
		Male	8.66 (2.77)	8.29 (2.56)
	− 1 + 2	Female	8.92 (2.78)	9.15 (2.49)
		Male	8.91 (2.88)	8.99 (3.18)

Because of the possibility that the effect of block order might have contributed to the presence of desirability effect in + 1‒2 condition and the absence of such an effect in ‒1 + 2 condition, we conducted a two-way mixed-design ANOVA of block order (Block 1 vs. Block 2) × Desirability–Block Order mapping (+ 1 − 2 vs. − 1 + 2). The main effect of block order was significant [*F*_(1,289)_ = 19.37, *p* < 0.001, *η*_*p*_^*2*^ = 0.06], showing that CoDG width was wider in Block 1 [*M* = 8.87, *SE* = 0.16] than that in Block 2 [*M* = 8.44, *SE* = 0.15]. The interaction between block order and desirability–block order mapping was also significant [*F*_(1,289)_ = 7.01, *p* = 0.009, *η*_*p*_^*2*^ = 0.02], showing that only in the condition of + 1 − 2, CoDG width was wider in Block 1 [*M* = 8.62, *SE* = 0.22] than that in Block 2 [*M* = 7.93, *SE* = 0.21].

### Discussion

In this experiment, we did not observe a main effect of trait desirability on CoDG width although the likeability rating results confirmed the effectiveness of the trait desirability manipulation. However, the desirability effect was moderated by desirability-block order mapping, with the effect emerging only in the + 1‒2 condition and not in the ‒1 + 2 condition. The results also showed an effect of block order; participants’ CoDG width was wider for the first identity (first block) than the second identity (second block). Thus, the interaction complicates the interpretation of the effect of trait desirability on CoDG width. Namely, it is possible that the observed desirability effect on CoDG width in the + 1‒2 condition reflected a training effect because enhanced perceptual sensitivity due to training could have caused CoDG width to become narrower in Block 2 versus Block 1. In the ‒1 + 2 condition, the training effect might have counteracted the widening effect by inferred desirable traits on CoDG width, thereby leading to no desirability effect on CoDG width. Additionally, consistent with findings from Experiment 1, men exhibited wider CoDG width than women. However, neither participant gender nor face sex moderated the desirability effect. Like in Experiment 1, the likeability rating showed participants’ preference for female faces.

## Experiment 3

In Experiment 2, narrowing of the gaze cone width from Block 1 to Block 2 (a training effect) was an unexpected result. To eliminate the block order effect that influenced the effect of trait desirability, in Experiment 3, trait desirability was treated as a between-subjects variable. To maintain consistency with the previous experiments, participants were still presented with two identities, but participants viewed an identity without any trait information in the first block, followed by either a likeable or an unlikeable identity of the same sex in the second block. The first trait-neutral identity served as a reference for comparing the second identity, whose trait desirability would be manipulated. The CoDG width for a likeable identity and an unlikeable identity was compared only based on the eye-contact judgment responses in the second block.

### Methods

#### Participants

We acquired 452 participants’ data in this experiment online via Prolific. The inclusion criteria were identical to those used in Experiments 1 and 2. Exclusion were applied as follows: in 35 participants the proportion of “yes” responses to averted gaze at 8˚exceeded 50% or the proportion of “yes” responses to direct gaze was below 50%; 53 participants were excluded due to poor model fit; two participants encountered technical issues (self-reported and data examination); and seven participants had CoDG values outside the range of mean ± 3 standard deviations. After these exclusions, the final sample consisted of 355 participants (179 women: *M*_Age_ = 39.14 years, *SD* = 11.74; 176 men: *M*_Age_ = 42.81 years, *SD* = 13.76; Ethnicity (White/Black/Asian/Mixed/Other): Men: 150/10/8/6/2; Women: 170/0/4/5/0). The age difference between the women and men was significant (*t*(353) = − 2.71, *p* = 0.007, *d* = − 0.29). Finally, we also checked participants’ responses on attention check trials (16 trials of 20˚ gaze direction), and the average error rate on the catch trials was 0.64%. One participant made five erroneous responses, three participants made two, 25 participants made one, and the remaining 326 participants made no erroneous responses.

#### Stimuli and procedure

This experiment consisted of four task versions for each face sex: (1) neutral F1/M1 (Block 1) and F2/M2 with desirable traits (Block 2); (2) neutral F1/M1 (Block 1) and F2/M2 with undesirable traits (Block 2); (3) neutral F2/M2 (Block 1) and F1/M1 with desirable traits (Block 2); (4) neutral F2/M2 (Block 1) and F1/M1 with undesirable traits (Block 2). The face stimuli and short vignettes used to manipulate trait desirability were identical to those used in Experiment 2. All participants began with the eye-contact task showing the face of an individual of whom they did not receive any trait information. After this, participants were presented with the face of the second individual whose traits were manipulated as either desirable or undesirable. This individual was of the same sex with the first individual. They then completed the impression-formation task, the trait-judgment task, and the eye-contact judgment task, all of which were identical to the tasks in Experiment 2. Finally, participants rated the likeability of the two identities, as in the previous experiments.

#### Data analysis

The procedures for data cleaning and calculation of CoDG width were identical to those used in Experiments 1 and 2. Multivariate ANOVA analyses were conducted with the following factors: 2 [Desirability of inferred traits: desirable vs. undesirable] × 2 [Participant gender: woman vs. man] × 2 [Face sex: female vs. male]. These analyses were performed on the gaze cone data and the likeability data for both the trait-neutral identities and trait-manipulated identities. Additionally, age was included as a covariate in the analysis of the gaze-cone data. For the gaze-cone width of both the trait-neutral identities [*F*_(1,346)_ = 39.40,* p* < 0.001, *η*_*p*_^*2*^ = 0.10] and trait manipulated identities [*F*_(1,346)_ = 14.40,* p* < 0.001, *η*_*p*_^*2*^ = 0.04], the effect of participants’ age as a covariate was significant, indicating that the older the participant, the wider the CoDG.^3^ Complete ANOVA results for trait-judgment and likeability ratings are provided in the Supplemental File (Table S11).

### Results

#### Likeability

We first examined participants’ likeability ratings for the identities without trait desirability manipulation across all subgroups of Desirability of Inferred Traits × Participant Gender × Face Sex. A significant main effect of face sex [*F*_(1,346)_ = 19.26,* p* < 0.001, *η*_*p*_^*2*^ = 0.05] indicated that female faces’ [*M* = 4.93, *SE* = 0.09] received higher likeability ratings compared with male faces [*M* = 4.36, *SE* = 0.09].

For the facial identities with trait desirability manipulation, a significant main effect of desirability [*F*_(1,346)_ = 247.80, *p* < 0.001, *η*_*p*_^*2*^ = 0.42] showed that identities associated with desirable traits [*M* = 5.27, *SE* = 0.10] were rated as more likeable compared with those associated with undesirable traits [*M* = 3.04, *SE* = 0.10]. Additionally, there was a significant interaction between desirability and face sex [*F*_(1,346)_ = 5.15, *p* = 0.024, *η*_*p*_^*2*^ = 0.02]. The effect of desirability was significant for both female faces and male faces [*p* values < 0.001], but it was greater for female faces [*M*_+_ = 5.56, *SE* = 0.14; *M*_−_ = 3.01, *SE* = 0.14] than male faces [*M*_+_ = 4.97, *SE* = 0.14; *M*_−_ = 3.07, *SE* = 0.15]. Further analyses showed that likeable female faces were rated as more likeable than likeable male faces [*p* = 0.002].^4^

#### The cone of direct gaze (CoDG)

**Fig. 4:**
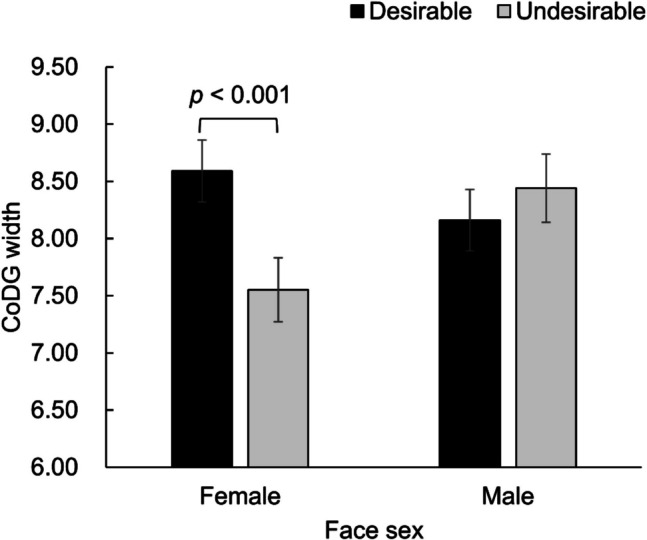
Means and standard errors of CoDG width as a function of desirability and face sex

For identities without trait manipulation, all the main effects and interactions were not significant [all *p* values > 0.05]. Table 3 shows the means (*SD*s) of CoDG width in each cell of Desirability of inferred Traits × Participant gender × Face sex for the identities with trait manipulation. For them, the main effect of desirability was not significant [*F*_(1,346)_ = 1.54,* p* = 0.215]. However, there was a significant interaction between desirability and face sex [*F*_(1,346)_ = 4.19,* p* = 0.041, *η*_*p*_^*2*^ = 0.01]. The breakdown of the interaction revealed that the CoDG width was significantly wider for likeable female faces [*M* = 8.55, *SE* = 0.27] compared with unlikeable female faces [*M* = 7.63, *SE* = 0.28], whereas no significant difference was observed in CoDG width for likeable male faces [*M* = 8.11, *SE* = 0.27] versus unlikeable male faces [*M* = 8.33, *SE* = 0.30] (see Fig. 4). The main effects of participant gender, face sex, and all other interactions were not significant [all *p* values > 0.05]. The results about the training effect on CoDG width can be seen in Supplemental File (Table S12).

**Table 3:** Means (and standard deviations) of CoDG width by participant gender, face sex, and desirability for the trait-manipulated identity

Participant gender	Face sex	Desirability	Mean (*SD*)
Man	Female	Desirable	9.05 (2.82)
		Undesirable	7.75 (2.89)
	Male	Desirable	8.16 (2.73)
		Undesirable	8.18 (2.27)
Woman	Female	Desirable	8.07 (2.48)
		Undesirable	7.35 (2.27)
	Male	Desirable	8.06 (2.71)
		Undesirable	8.64 (2.92)

### Discussion

In this experiment, the gaze cone data did not show a main effect of trait desirability on eye contact perception, although the likeability rating results confirmed the effectiveness of the trait desirability manipulation. However, the effect of trait desirability on CoDG width was moderated by face sex. The desirability effect was observed exclusively for female faces, revealing that the CoDG width was wider likeable female faces as compared with unlikeable female faces. It is also important to note that, regardless of face sex, the CoDG width for trait-neutral identities did not differ when they preceded likeable faces versus unlikeable faces. Thus, the difference in CoDG width between likeable versus unlikeable female faces resulted from the trait desirability manipulation. Consistent with Experiments 1 and 2, a preference for female faces over male faces was observed, both when no trait information was provided and when the faces were described as likeable.

## General discussion

The present study aimed to investigate how trait inference, based on other people’s behaviors varying in social desirability, influences the perception of their direct gaze, as measured by the cone of direct gaze (CoDG). Additionally, we investigated whether participant gender and stimulus face sex modulate the effect of inferred trait desirability on CoDG width. To achieve this, participants first completed an impression-formation task in which they read verbal descriptions of social behaviors to infer individuals’ trait desirability. Subsequently, in an eye-contact judgment task, they judged whether the individuals portrayed with either direct gaze or slightly averted gaze (gaze directions deviating from direct gaze by 2˚, 4˚, 6˚, or 8˚) were looking at them or not. In Experiment 1, participants viewed either a likeable female face and an unlikeable male face or vice versa. In Experiment 2, participants viewed two faces of the same sex, either beginning with a likeable face followed by an unlikeable face or vice versa. Finally, in Experiment 3, participants viewed two faces of the same sex, with a trait-neutral face first followed by either a likeable or an unlikeable face.

At the end of each experiment, we assessed whether participants kept the initial impressions of the individuals by asking them to evaluate the individuals’ likeability. The likeability ratings indicated that individuals described with socially desirable behaviors were evaluated as more likeable than those described with socially undesirable behaviors. These findings demonstrate that participants acquired trait knowledge about the individuals in the impression-formation task (as also confirmed by the trait-judgment task; see the Supplemental File) and that the formed impressions were durable.

Overall, the results showed that it was exclusively the trait desirability of female gazers that moderated participants’ CoDG width, indicating that participants CoDG width was wider when seeing likeable female faces as opposed to unlikeable female faces. Participants were more likely to judge a likeable female person as making eye contact with them than the unlikeable female person. This finding was novel but not entirely unexpected, as face sex may affect the perception and evaluation of social partners, particularly when these partners are associated with traits varying in social desirability. Stereotypical women are generally perceived more positively than stereotypical men because women are often associated with higher levels of femininity and prosocial traits (Eagly & Mladinic, 1994), such as warmth (Delacollette et al., 2013), trustworthiness (Wirth & Wentura, 2023), and concern for others (Williams & Best, 1982). However, women with counterstereotypical facial features (e.g., masculine) or traits (e.g., dominance or untrustworthiness) are evaluated more negatively compared with stereotypical men and women (Sutherland et al., 2015). In contrast, men with counterstereotypical facial features (e.g., feminine) or traits (e.g., nondominance) tend to be evaluated as positively as stereotypical men and women (Sutherland et al., 2015). Thus, the effect of social desirability manipulation on eye contact perception was more likely to emerge for female faces.

Participants may already expect female faces to display socially desirable traits. The verbal descriptions of social behaviors in the task, which were congruent with these expectations, likely reinforced the stereotypes about female faces. This might have amplified the overall positive evaluations of the female faces (a halo effect), increasing participants’ liking for them. Supporting this, the likeability rating task showed that participants rated likeable female faces as more likeable than likeable male faces. Therefore, it was more likely that participants felt a socially desirable female person making eye contact with them compared with an undesirable female person. Although participants could infer the traits of male persons from verbal descriptions of their social behaviors, men may also be stereotypically and automatically evaluated as dominant. As a result, manipulating social desirability might not significantly influence the perception of eye contact with male faces, as participants may have been influenced by existing gender stereotypes about men.

Participants’ CoDG width may reflect their prepared action tendency elicited by trait inference. It has been suggested that trait inference serves an action preparation function, motivating the observer to approach or avoid a given individual, which may influence the likelihood of future interactions (Crawford et al., 2013; McCarthy & Skowronski, 2011). Thus, a widened CoDG when seeing a person associated with desirable traits reflects an approach-related motivation tendency. As direct gaze has been interpreted to indicate the sender’s tendency of approach (Adams & Kleck, 2003, 2005), the perceiver’s wide CoDG reassures them that the likeable person is a potential target worth interacting with. Conversely, a narrowed CoDG when seeing a person with undesirable traits manifests the observer’s motivational tendency of avoidance. Since another person not making eye contact (i.e., averted gaze) signals disinterest or avoidance tendency, the observer’s narrowed CoDG, caused by their own avoidance tendency, leads to a decreased likelihood of initiating interaction.

Alternatively, but not mutually exclusively, the widened gaze cone can be interpreted as a manifestation of self-referential positivity bias, wherein individuals prioritize self-related processing and tend to evaluate themselves more positively than others (Pahl & Eiser, 2005). Observers are more likely to interpret that someone conveying positive social signals, such as a smile, are making eye contact with them (Gianotti et al., 2018; Lobmaier et al., 2008; Lobmaier & Perrett, 2011). Being looked at by such a person aligns with, or even reinforces, the belief that the observers themselves are positive and likeable. Moreover, one study has directly demonstrated that the CoDG width was wider for others’ positive versus negative utterances, but only when the speech was directed at the self (Chen & Hietanen, 2024). In the present study, participants might have judged, because of the self-referential positivity bias, that people behaving in a socially desirable manner were making eye contact with them—that is, they deserved that a likeable person was directing their attention (interest) towards them.

Across three experiments, we consistently observed significant effects of age on eye contact perception, indicating that older participants exhibited a wider CoDG. As the present study did not aim to investigate the effects of age on eye-contact perception, we acknowledge that explaining this ageing effect is challenging without additional age-relevant information. However, the findings are unlikely to be spurious, as they replicate the results by Hietanen et al. (2022) and Lasagna et al. (2020; but the ageing effect did not survive the correction for multiple comparisons). In the study by Hietanen et al. (2022), besides the effect of age on CoDG width, the results also revealed that, compared with younger adults, older adults had a greater tendency toward self-referential processing. The authors suggested that as older adults also tend to focus more on positive than negative information, a well-documented phenomenon known as the positivity shift (Mather & Carstensen, 2005), the wider gaze cone observed in older adults may reflect an enhanced self-referential positivity bias. That is, because direct gaze is an affiliative and inclusive signal that can elicit positive affective responses, the increased tendency for self-referential processing may lead older adults to perceive others’ gaze as directed them, even when the gaze is slightly averted.

The results also showed that, in Experiments 1 and 2, men had a wider CoDG than women, which is consistent with previous studies (Hietanen et al., 2022; Huang et al., 2022; Lobmaier et al., 2008; Zhai et al., 2025). One explanation is that women may be more sensitive to direct gaze than men (Lasagna et al., 2020), and the narrower CoDG in women reflects more accurate discrimination between direct and slightly averted gaze (Huang et al., 2022; Lobmaier et al., 2008). Another possible explanation concerns gender differences in self-construal. Compared with women, whose interdependent construal leads them to prioritize connection and harmony in relationships, men’s independent self-construal leads them to focus more on themselves (Cross & Madson, 1997; Guimond et al., 2006; Kashima et al., 2004; Traut-Mattausch et al., 2011). As a result, men may be more likely to perceive another’s slightly averted gaze as directed at them, which is reflected in a wider CoDG (Huang et al., 2022). However, we cannot know with certainty whether other participant-related factors also contributed to the gender difference in CoDG width, especially since Experiment 3 did not show such a difference. Therefore, we cannot draw firm conclusions about the mechanisms underlying this gender difference.

In Experiment 1, participants’ gender qualified the effect of desirability on CoDG width. While men’s CoDG width did not differ significantly between seeing a likeable person versus an unlikeable person, women exhibited wider CoDG width when seeing a likeable person compared with an unlikeable person. We tentatively interpret this gender difference in terms of how individuals perceive the self and how they respond to unlikeable people. Regardless of gender, participants might have judged that people behaving in a socially desirable manner were making eye contact with them because of the self-referential positivity bias—that is, they deserved that a likeable person was directing their attention (interest) towards them. However, gender differences may result from how men and women respond to individuals behaving in a socially undesirable way. Men, with an independent self-construal, often use relationships with others as a mirror for social comparison, assessing themselves relative to others, whereas women, with an interdependent self-construal, tend to prioritize connection and harmony in relationships (Cross & Madson, 1997; Guimond et al., 2006; Kashima et al., 2004; Traut-Mattausch et al., 2011). By comparing themselves with a socially undesirable counterpart, men may emphasize their superiority and boost their self-esteem. In contrast, women may focus less on a socially unlikeable person and more on recognizing potential interactants, developing new relationships and maintaining connectedness with likeable individuals. Therefore, in women, the self-referential positivity bias or the tendency to prioritize relationships and connection, or both, could widen the CoDG width when seeing likeable people. In men, the self-referential positivity bias might widen the CoDG width when seeing likeable people. However, the inferred undesirable traits can serve as a source of social comparison, potentially enhancing self-esteem, and thus also leading to widened CoDG width. Thus, no significant difference in CoDG width was observed for men when seeing likeable versus unlikeable people.

Why was this result not replicated in Experiments 2 and 3? The inconsistency might stem from differences in the face stimuli and the experimental designs. In Experiment 1, a female face and a male face were presented in the impression-formation task and the eye-contact judgment task. This design might have provided men and women with opportunities to compare themselves with a likeable or unlikeable opposite-sex gazer. Opposite-sex social comparison could further encourage participants to rely on attributes shared by their gender group to define themselves. In such cases, women’s self-construal as interdependent and men’s self-construal as independent were emphasized (Guimond et al., 2006; Onorato & Turner, 2004). Thus, as we addressed, the gender difference in self-construal might explain why the effect of trait desirability on eye contact perception was moderated by participant gender. In Experiments 2 and 3, same-sex faces were used, and they were presented in different blocks. This temporal separation might have led participants to focus solely on the contrast in inferred trait desirability between the two same-sex identities when encountering the second person. We believe that when participants encountered the second person, they tended not to compare themselves with the gazers. Consequently, the moderating role of participant gender in judging eye contact with likeable or unlikeable individuals did not emerge in Experiments 2 and 3. However, these interpretations remain speculative and await future research’s justification.

## Limitation, future directions, and conclusions

A limitation of our study is that it lacked a control condition using faces associated with inferred neutral traits. In Experiment 3, a neutral identity was presented, but we could not include it as a control condition due to the observed training effect on CoDG width. We discussed all the results in the light of inferred socially desirable traits widening CoDG width. Notwithstanding, the difference in the CoDG width between seeing likeable female faces versus unlikeable female faces is possibly due to the narrowing effect of inferred undesirable traits, or both the widening effect of inferred desirable traits and the narrowing effect of inferred undesirable traits. Future research is needed to compare the effects of desirable/undesirable traits with effects of neutral traits on CoDG width. Systematic comparisons of these alternatives could confirm whether only faces of persons associated with desirable traits widen CoDG width. Another limitation is that participants were only recruited from the UK. One may argue that individuals from Western, Educated, Industrialized, Rich, and Democratic (WEIRD) societies are not representative of the vast majority of the world’s population (Henrich et al., 2010). Future research on eye-contact perception would benefit from investigating participants (and stimulus faces) with a larger variation of cultural backgrounds. Additionally, it should be noted that we only recruited participants who identified their gender as binary (man/woman). Future research should address how non-binary individuals perceive and interpret gendered social information and how this influences their eye-contact perception. Another point is that the faces in our study were easily identifiable as female or male, and their social behaviors were described using gendered language (e.g., She/He). Thus, another future direction should explore how eye gaze information displayed by faces of non-binary or androgynous faces is perceived by others.

In conclusion, the present study demonstrated that trait inference based on descriptions of women’s social behavior affected observers’ perception of direct gaze, as measured by CoDG width. Observes exhibited a wider CoDG for likeable women compared with unlikeable ones. These findings highlight how individuals’ gender-related stereotypes, combined with a social partner’s traits of varying social desirability, affect perceivers’ perception of their partner’s facial cues. Desirable traits inferred from women’s social behaviors may align with individuals’ expectations of stereotypical women, enhancing their perceived likeability. Conversely, undesirable traits inferred from women’s social behaviors may violate these expectations. We propose that the mechanism underlying the widened CoDG width by women with desirable traits is individuals’ self-referential positivity bias or the approach-related action tendency elicited by desirable traits, or a combination of both. As far as we know, the present study is the first to empirically show that trait inference by reading trait-implied behaviors affects the perception of direct gaze. Moreover, the findings resonate with previous research proposing that individuals’ traits can elicit supportive or unsupportive reactions in perceivers (Pierce et al., 1997; Barańczuk, 2019), which, in turn, leads observers to approach or avoid them, respectively. In the present study, this was reflected in observers’ CoDG width.

## Supplementary Information

Below is the link to the electronic supplementary material.

Supplementary file1 (DOCX 69 KB)

## Data Availability

The data and all the experimental materials are available in the Open Science Framework (https://osf.io/er2pb/).

## Footnotes

1 The main results did not change when age was not included as a covariate: the main effects of desirability [*F*_(1,346)_ = 6.19, *p* =.013, *η*_*p*_^*2*^ = 0.02] and participant gender [*F*_(1,346)_ = 6.90,* p* =.009, *η*_*p*_^*2*^ = 0.02], as well as the interactions of desirability × participant gender [*F*_(1,346)_ = 6.23, *p* =.013, *η*_*p*_^*2*^ = 0.02] and desirability × desirability–face sex mapping [*F*_(1,346)_ = 9.09,* p* = 0.003, *η*_*p*_^*2*^ = 0.03] were significant. More details can be found in the Supplemental File (Table S4).

2 When age was not included as a covariate, in addition to the significant main effect of desirability–block order mapping [*F*_(1,283)_ = 5.75, *p* =.017, *η*_*p*_^*2*^ = 0.02] as well as the significant interactions of desirability × desirability–block order mapping [*F*_(1,283)_ = 20.01, *p* <.001, *η*_*p*_^*2*^ = 0.07] and face sex × desirability–block order mapping [*F*_(1,283)_ = 5.08, *p* =.025, *η*_*p*_^*2*^ = 0.02] reported in the main text, the main effect of desirability [*F*_(1,283)_ = 7.44, *p* =.007, *η*_*p*_^*2*^ = 0.03] was also significant. However, the previously reported significant interaction of participant gender × desirability–block order mapping [*F*_(1,283)_ = 3.56, *p* =.06] was not significant. More details can be found in the Supplemental File (Table S7).

3 The main results did not change when age was not included as a covariate. For identities without trait manipulation, all the main effects and interactions were not significant [all *p* values > 0.05]. For the identities with trait manipulation, as reported in the main text, only the interaction of desirability × face sex [*F*_(1,347)_ = 5.38, *p* = 0.021, *η*_*p*_^*2*^ = 0.02] was significant. More details can be found in the Supplemental File (Tables S10).

4 One participant whose data were included in the analyses in the eye-contact judgment task and trait-judgment task was not included in the likeability rating task because the rating was missing perhaps due to technical issues.

## Code availability

No custom code was used in the data analysis.

## Declarations

### Open practicesEthics approval

The principles outlined in the Declaration of Helsinki were followed when conducting this research. The Ethics Committee of Tampere region approved that the present study was exempt from a formal ethical review and evaluated that the present study followed good scientific practice before the submission, and a statement regarding their decision was obtained (Date: 04/06/2024).

Consent to participate: Informed consent for participation was obtained from all individual participants in the study.

### Open practices

The data and materials for this study are available (https://osf.io/er2pb/) and the experiment was not preregistered.
