# Supplementary material for: Inferences about women’s traits influence the judgment of their eye contact
Source: Psychon Bull Rev. 2025 May 27;32(6):2860–77. doi: 10.3758/s13423-025-02704-7 (PMC12627109; doi:10.3758/s13423-025-02704-7)
Supplement: Supplementary file 1 — Supplementary file1 (DOCX 69 KB) [file 13423_2025_2704_MOESM1_ESM.docx]

Supplemental Materials

Inferences about women’s traits influence the judgment of their eye contact

Manman Zhai and Jari K. Hietanen

Tampere University

Author Note

Manman Zhai (0000-0002-6305-8715) and Jari Hietanen (0000-0002-7585-3775), Human Information Processing Laboratory, Faculty of Social Sciences / Psychology, Tampere University.

Correspondence concerning this article should be addressed to Jari Hietanen, Human Information Processing Laboratory, Faculty of Social Sciences / Psychology, FI-33014 Tampere University, Tampere, Finland. Telephone number: +358-40-190 1384. E-mail: [jari.hietanen@tuni.fi](mailto:jari.hietanen@tuni.fi).

**Experiment 1 Supplemental Tables and Analyses**

**Table S1 The means (and standard deviations) of valence, sociability, and observability ratings for trait adjectives.**

| Word_English | Valence Mean (SD) | Sociability Mean (SD) | Observability Mean (SD) |
| --- | --- | --- | --- |
| caring | 2.35 (0.80) | 2.47 (0.78) | 3.22 (0.72) |
| friendly | 2.32 (0.83) | 2.5 (0.82) | 3.56 (0.55) |
| generous | 2.29 (0.83) | 2.48 (0.77) | 3.16 (0.81) |
| helpful | 2.36 (0.77) | 2.55 (0.72) | 3.37 (0.68) |
| reliable | 2.43 (0.76) | 2.49 (0.81) | 3.05 (0.95) |
|  |  |  |  |
| arrogant | -2.17 (0.95) | -2.12 (1.04) | 3.37 (0.81) |
| dishonest | -2.55 (0.65) | -2.41 (1.23) | 2.36 (1.05) |
| hateful | -2.64 (0.64) | -2.44 (1.17) | 3.01 (0.98) |
| rude | -2.34 (0.82) | -2.31 (1.14) | 3.47 (0.84) |
| violent | -2.59 (0.94) | -2.66 (0.88) | 3.31 (0.94) |

Note:

For the valence ratings, participants were asked to rate each word with respect to the emotional meaning of the trait in terms of its degree of positivity or negativity.

For the sociality ratings, participants were asked to indicate the degree of social desirability they associated with each word.

For the observability ratings, participants were asked to rate each word for how observable the trait was to other people (Britz et al., 2022).

**Table S2** **Trait-implied words and sentences describing corresponding social behaviors.**

| Words | Sentences |
| --- | --- |
| caring | She/He always listens attentively to others’ feelings and problems. |
| friendly | She/He greeted everyone with a smile and a compliment at the party. |
| generous | She/He donates to various causes and charities. |
| helpful | She/He helped an old lady cross the street. |
| reliable | She/He always keeps his promises and never lets anyone down. |
|  |  |
| arrogant | She/He always thinks she/he is better than everyone else and never listens to other people’s opinions. |
| dishonest | She/He lies and cheats to get what he wants. |
| hateful | She/He blames and complains about everything and everyone. |
| rude | She/He interrupts and talks over others without listening. |
| violent | She/He throws things and breaks them when she is angry. |

**Table S3** **The statistics of attractiveness ratings and comparisons of attractiveness ratings between men and women for each stimulus face identity (F for female faces, M for male faces).**

| Stimulus | Participant’s gender | Mean | Std. Deviation | Sig. | 95% confidence interval for difference |
| --- | --- | --- | --- | --- | --- |
| F1 | Woman | 6.50 | 1.43 | 0.74 | [-0.61, 0.85] |
|  | Man | 6.38 | 1.35 |  |  |
| F2 | Woman | 6.23 | 1.41 | 0.88 | [-0.71, 0.84] |
|  | Man | 6.17 | 1.56 |  |  |
| M1 | Woman | 5.00 | 1.55 | 0.42 | [-0.46, 1.08] |
|  | Man | 4.69 | 1.39 |  |  |
| M2 | Woman | 5.20 | 1.92 | 0.99 | [-0.91, 0.90] |
|  | Man | 5.21 | 1.54 |  |  |

**Table S4 Analysis of variance (ANOVA) results on CoDG width in Experiment 1 without age included as a covariate.**

| Source | F | df | Sig. | *η_p_*^2^ |
| --- | --- | --- | --- | --- |
| Desirability (D) | 6.190* | 1, 346 | 0.013 | 0.018 |
| Participant gender (G) | 6.899** | 1, 346 | 0.009 | 0.020 |
| Desirability-face sex mapping (D-S) | 1.923 | 1, 346 | 0.166 | 0.006 |
| D × G | 6.230* | 1, 346 | 0.013 | 0.018 |
| D × D-S | 9.091** | 1, 346 | 0.003 | 0.026 |
| G × D-S | 0.067 | 1, 346 | 0.796 | 0.000 |
| D × G × D-S | 0.118 | 1, 346 | 0.731 | 0.000 |

**Table S5** **Analysis of variance (ANOVA) results on ratings of trait judgment and likeability in Experiment 1.**

| Task | | Source | F | df | Sig. | *η_p_*^2^ |
| --- | --- | --- | --- | --- | --- | --- |
| Trait judgment check | Words implying desirable traits | Desirability (D) | 2257.51*** | 1, 346 | < 0.001 | 0.867 |
|  |  | Participant gender (G) | 0.39 | 1, 346 | 0.532 | 0.001 |
|  |  | Desirability-face sex mapping (D-S) | 3.36 | 1, 346 | 0.068 | 0.010 |
|  |  | D × G | 0.12 | 1, 346 | 0.728 | 0.000 |
|  |  | D × D-S | 0.30 | 1, 346 | 0.585 | 0.001 |
|  |  | G × D-S | 0.82 | 1, 346 | 0.367 | 0.002 |
|  |  | D × G × D-S | 0.37 | 1, 346 | 0.545 | 0.001 |
|  | Words implying undesirable traits | Desirability (D) | 2185.64*** | 1, 346 | < 0.001 | 0.863 |
|  |  | Participant gender (G) | 3.43 | 1, 346 | 0.065 | 0.010 |
|  |  | Desirability-face sex mapping (D-S) | 7.63** | 1, 346 | 0.006 | 0.022 |
|  |  | D × G | 0.97 | 1, 346 | 0.326 | 0.003 |
|  |  | D × D-S | 5.69* | 1, 346 | 0.018 | 0.016 |
|  |  | G × D-S | 1.21 | 1, 346 | 0.273 | 0.003 |
|  |  | D × G × D-S | 0.83 | 1, 346 | 0.364 | 0.002 |
| Likeability rating | | Desirability (D) | 196.48*** | 1, 346 | < 0.001 | 0.362 |
|  |  | Participant gender (G) | 8.59** | 1, 346 | 0.004 | 0.024 |
|  |  | Desirability-face sex mapping (D-S) | 0.17 | 1, 346 | 0.682 | 0.000 |
|  |  | D × G | 0.50 | 1, 346 | 0.481 | 0.001 |
|  |  | D × D-S | 39.42*** | 1, 346 | < 0.001 | 0.102 |
|  |  | G × D-S | 0.00 | 1, 346 | 0.996 | 0.000 |
|  |  | D × G × D-S | 3.82 | 1, 346 | 0.052 | 0.011 |

**Supplemental Analysis 1.** **Method, Results, and Discussions of the trait judgment check in Experiment 1.**

For the trait judgment task, we calculated two scores for each of the 350 participants by summing their ratings (on a 1-7 scale) of how well the five adjectives implying socially desirable traits and five adjectives implying socially undesirable traits described the likable person and unlikeable person.

For words implying desirable traits, a significant main effect of desirability [*F*_(1,346)_ = 2257.51, *p* < 0.001, *η_p_*^2^ = 0.87] showed that these words were rated as better describing the likeable people [*M* = 31.17, *SE* = 0.28] than the unlikeable people [*M* = 8.70, *SE* = 0.25].

For words implying undesirable traits, a significant main effect of desirability [*F*_(1,346)_ = 2185.64, *p* < 0.001, *η_p_*^2^ = 0.86] showed that these words were rated as better describing the unlikeable people [*M* = 28.57, *SE* = 0.33] compared to the likeable people [*M* = 6.98, *SE* = 0.22]. There was also a significant interaction between desirability and desirability-face sex mapping [*F*_(1,346)_ = 5.69, *p* = 0.018, *η_p_*^2^ = 0.02]. The effect of desirability was significant for both pairs [*p*s < 0.001], but it was greater for the +F‒M pairs [*M*_+F_ = 6.85, *SE* = 0.30; *M*_‒M_ = 29.54, *SE* = 0.46] than the +M‒F pairs [*M*_+M_ = 7.11, *SE* = 0.31; *M*_‒F_ = 27.60, *SE* = 0.47]. Further analyses showed that the words implying undesirable traits were rated as better describing unlikeable male faces compared to unlikeable female faces [*p* = 0.003].

The results of trait judgment checks showed that the words implying socially desirable behaviors were evaluated to better describe the likeable people than the unlikeable people. Conversely, words implying socially undesirable behaviors were evaluated to better describe the unlikeable people than the likeable people. Additionally, a preference for female faces was observed in the trait judgment task. When asked to assess how well trait words described the faces, participants were more likely to associate undesirable trait adjectives with unlikeable male faces than with unlikeable female faces.

**Experiment 2 Supplemental Tables and Analyses**

**Table S6** **Trait-implied words and vignettes describing corresponding social behaviors.**

| Words | vignettes |
| --- | --- |
| caring | Imagine you were stressed about work. She/He noticed your mood and invited you over for coffee, listened without judgement, and gave advice when asked. Most importantly, she/he was there to make sure you felt understood and supported. |
| friendly | Imagine you were at a party where you knew no one. She/He greeted you with a warm smile and invited you to join her/him. She/He introduced you to others, making you feel welcome and included. |
| generous | Imagine you were struggling to pay for a class. She/He quietly transferred the money to cover it. She/He even offered to drive you each week, saving you the time of commuting, so you could focus on learning. |
| helpful | Imagine you had an injured ankle and were struggling to get around. She/He immediately drove you to your appointments and picked up groceries for you. She/He checked in daily, always ready to lend a hand if you needed anything. |
| reliable | Imagine you were sick. Despite the heavy rain, she/he showed up at your door, just as she/he promised, to take you to the doctor’s appointment. You knew you could count on her/him, no matter what. |
|  |  |
| arrogant | Imagine you participated in a group project. She/He constantly dismissed your ideas without considering them, acting like only her/his opinions mattered. Whenever you tried to contribute, she/he talked over you, making it clear she/he thought she/he knew better than everyone else. |
| dishonest | Imagine you worked on a report in a group. She/He claimed she/he had completed her/his part, but when it was time to submit, you discovered she/he hadn’t done anything. Instead of admitting it, she/he lied and blamed others, leaving you to fix everything at the last minute. |
| hateful | Imagine you were sharing your thoughts in a group discussion. She/He interrupted with harsh comments, attacking your perspective. Her/His cold tone made you feel uncomfortable and unwelcome, as if she/he took pleasure in putting you down. |
| rude | Imagine you were talking about your weekend plans. She/He interrupted, saying they sounded boring, and, without paying attention to how you felt, she/he started sharing her/his own plans instead. |
| violent | Imagine you shared an apartment with this person. One evening, she/he slipped and fell in the living room. Believing you had spilled tea on the floor, she/he became angry, shouting loudly and throwing the magazines from the coffee table onto the floor. |

**Table S7 Analysis of variance (ANOVA) results on CoDG width in Experiment 2 without age included as a covariate.**

| Source | F | df | Sig. | *η_p_*^2^ |
| --- | --- | --- | --- | --- |
| Desirability (D) | 7.435** | 1, 283 | 0.007 | 0.026 |
| Participant gender (G) | 4.520* | 1, 283 | 0.034 | 0.016 |
| Face sex (S) | 2.754 | 1, 283 | 0.098 | 0.010 |
| Desirability-block order mapping (D-BO) | 5.753* | 1, 283 | 0.017 | 0.020 |
| D × G | 0.222 | 1, 283 | 0.638 | 0.001 |
| D × S | 0.065 | 1, 283 | 0.799 | 0.000 |
| D × D-BO | 20.013*** | 1, 283 | 0.000 | 0.066 |
| G × S | 0.468 | 1, 283 | 0.495 | 0.002 |
| G × D-BO | 3.562 | 1, 283 | 0.060 | 0.012 |
| S × D-BO | 5.083* | 1, 283 | 0.025 | 0.018 |
| D × G × S | 1.113 | 1, 283 | 0.292 | 0.004 |
| D × G × D-BO | 0.460 | 1, 283 | 0.498 | 0.002 |
| D × S × D-BO | 0.118 | 1, 283 | 0.732 | 0.000 |
| G × S × D-BO | 0.159 | 1, 283 | 0.691 | 0.001 |
| D × G × S × D-BO | 3.465 | 1, 283 | 0.064 | 0.012 |

**Table S8** **Analysis of variance (ANOVA) results on ratings of trait judgment and likeability in Experiment 2.**

| Task | | Source | F | df | Sig. | *η_p_^2^* |
| --- | --- | --- | --- | --- | --- | --- |
| Trait judgment check | Words implying desirable traits | Desirability (D) | 8805.75*** | 1, 283 | < 0.001 | 0.969 |
|  |  | Participant gender (G) | 0.17 | 1, 283 | 0.678 | 0.001 |
|  |  | Face sex (S) | 2.32 | 1, 283 | 0.129 | 0.008 |
|  |  | Desirability-block order mapping  (D-BO) | 6.64* | 1, 283 | 0.010 | 0.023 |
|  |  | D × G | 7.62** | 1, 283 | 0.006 | 0.026 |
|  |  | D × S | 0.61 | 1, 283 | 0.435 | 0.002 |
|  |  | D × D-BO | 1.35 | 1, 283 | 0.246 | 0.005 |
|  |  | G × S | 1.99 | 1, 283 | 0.159 | 0.007 |
|  |  | G × D-BO | 1.05 | 1, 283 | 0.306 | 0.004 |
|  |  | S × D-BO | 1.74 | 1, 283 | 0.188 | 0.006 |
|  |  | D × G × S | 0.58 | 1, 283 | 0.447 | 0.002 |
|  |  | D × G × D-BO | 1.19 | 1, 283 | 0.276 | 0.004 |
|  |  | D × S × D-BO | 0.00 | 1, 283 | 0.958 | 0.000 |
|  |  | G × S × D-BO | 0.04 | 1, 283 | 0.846 | 0.000 |
|  |  | D × G × S × D-BO | 0.11 | 1, 283 | 0.736 | 0.000 |
|  | Words implying undesirable traits | Desirability (D) | 5908.12*** | 1, 283 | < 0.001 | 0.954 |
|  |  | Participant gender (G) | 0.86 | 1, 283 | 0.356 | 0.003 |
|  |  | Face sex (S) | 10.85** | 1, 283 | 0.001 | 0.037 |
|  |  | Desirability-block order mapping  (D-BO) | 0.54 | 1, 283 | 0.464 | 0.002 |
|  |  | D × G | 5.43* | 1, 283 | 0.021 | 0.019 |
|  |  | D × S | 1.14 | 1, 283 | 0.287 | 0.004 |
|  |  | D × D-BO | 0.05 | 1, 283 | 0.819 | 0.000 |
|  |  | G × S | 1.97 | 1, 283 | 0.161 | 0.007 |
|  |  | G × D-BO | 0.07 | 1, 283 | 0.786 | 0.000 |
|  |  | S × D-BO | 0.01 | 1, 283 | 0.917 | 0.000 |
|  |  | D × G × S | 0.01 | 1, 283 | 0.937 | 0.000 |
|  |  | D × G × D-BO | 2.05 | 1, 283 | 0.153 | 0.007 |
|  |  | D × S × D-BO | 1.92 | 1, 283 | 0.167 | 0.007 |
|  |  | G × S × D-BO | 0.02 | 1, 283 | 0.875 | 0.000 |
|  |  | D × G × S × D-BO | 0.44 | 1, 283 | 0.505 | 0.002 |
| Likeability rating | | Desirability (D) | 381.62*** | 1, 283 | < 0.001 | 0.574 |
|  |  | Participant gender (G) | 0.27 | 1, 283 | 0.607 | 0.001 |
|  |  | Face sex (S) | 20.62*** | 1, 283 | < 0.001 | 0.068 |
|  |  | Desirability-block order mapping  (D-BO) | 2.21 | 1, 283 | 0.138 | 0.008 |
|  |  | D × G | 0.08 | 1, 283 | 0.772 | 0.000 |
|  |  | D × S | 0.79 | 1, 283 | 0.373 | 0.003 |
|  |  | D × D-BO | 2.91 | 1, 283 | 0.089 | 0.010 |
|  |  | G × S | 0.85 | 1, 283 | 0.358 | 0.003 |
|  |  | G × D-BO | 0.18 | 1, 283 | 0.674 | 0.001 |
|  |  | S × D-BO | 2.59 | 1, 283 | 0.109 | 0.009 |
|  |  | D × G × S | 1.07 | 1, 283 | 0.301 | 0.004 |
|  |  | D × G × D-BO | 0.02 | 1, 283 | 0.888 | 0.000 |
|  |  | D × S × D-BO | 0.20 | 1, 283 | 0.659 | 0.001 |
|  |  | G × S × D-BO | 0.57 | 1, 283 | 0.451 | 0.002 |
|  |  | D × G × S × D-BO | 0.01 | 1, 283 | 0.940 | 0.000 |

**Supplemental Analysis 2.** **Discussions of the trait judgment check in Experiment 2**

For words implying desirable traits, a significant main effect of desirability [*F*_(1,283)_ = 8805.75, *p* < 0.001, *η_p_*^2^ = 0.97] showed that these words were rated as better describing the likeable people [*M* = 33.42, *SE* = 0.20] than the unlikeable people [*M* = 6.49, *SE* = 0.17]. There was also a significant interaction between desirability and participant gender [*F*_(1,283)_ = 7.62, *p* = 0.006, *η_p_*^2^ = 0.03]. The effect of desirability was significant for both men and women [*p*s < 0.001], but it was greater for women [*M*_+_ = 33.86, *SE* = 0.28; *M*_‒_ = 6.14, *SE* = 0.23] than men [*M*_+_ = 32.98, *SE* = 0.28; *M*_‒_ = 6.84, *SE* = 0.24]. Further analyses showed that women rated these words as more descriptive of likeable faces [*p* = 0.026] and as less descriptive of unlikeable faces [*p* = 0.036] compared to men.

For words implying undesirable traits, a significant main effect of desirability [*F*_(1,283)_ = 5908.13, *p* < 0.001, *η_p_*^2^ = 0.95] showed that these words were rated as better describing the unlikeable people [*M* = 29.51, *SE* = 0.24] than the likeable people [*M* = 5.97, *SE* = 0.15]. A significant main effect of face sex [*F*_(1,283)_ = 10.85, *p* = 0.001, *η_p_*^2^ = 0.04] indicated that the words were rated as better describing male faces [*M* = 18.15, *SE* = 0.19] than female faces [*M* = 17.33, *SE* = 0.17]. Interestingly, there was a significant interaction between desirability and participant gender [*F*_(1,283)_ = 5.43, *p* = 0.021, *η_p_*^2^ = 0.02]. The effect of desirability was significant for both men and women [*p*s < 0.001], but it was greater for women [*M*_+_ = 5.73, *SE* = 0.21; *M*_‒_ = 29.98, *SE* = 0.33] than men [*M*_+_ = 6.21, *SE* = 0.21; *M*_‒_ = 29.04, *SE* = 0.34]. Further analyses showed that women rated these words as better describing unlikeable faces as compared to men.

Compatible with Experiment 1, the results showed the effectiveness of the trait desirability manipulation. The results also showed a preference for female faces, as participants were less willing to associate words implying undesirable traits with female faces. Additionally, participants’ gender had an effect on trait judgments: compared to men, women were more likely to associate desirable traits with likeable people and undesirable traits with unlikeable people, while being less likely to associate desirable traits with unlikeable people. This pattern suggests that women may have been more sensitive to the desirability of others’ traits than men, a sensitivity that is important in establishing and maintaining social connectedness.

**Table S9 The interaction of Desirability-block order mapping with Face sex and Participant gender on CoDG width in Experiment 2.**

| Interaction | F | df | Sig. | *η_p_^2^* | Desirability-block order mapping | Face sex | Mean | SE | Sig.  (Pairwise comparison) |
| --- | --- | --- | --- | --- | --- | --- | --- | --- | --- |
| Desirability-block order mapping × Face sex | 4.54 | 1, 282 | 0.034 | 0.02 | +1-2 | Female | 7.78 | 0.26 | 0.01 |
|  |  |  |  |  |  | Male | 8.88 | 0.30 |  |
|  |  |  |  |  | -1+2 | Female | 9.08 | 0.26 | 0.83 |
|  |  |  |  |  |  | Male | 8.99 | 0.29 |  |
|  |  |  |  |  |  | Participant gender | Mean | SE | Sig. |
| Desirability-block order mapping × Participant gender | 4.36 | 1, 282 | 0.038 | 0.02 | +1-2 | Man | 8.90 | 0.28 | 0.005 |
|  |  |  |  |  |  | Woman | 7.77 | 0.28 |  |
|  |  |  |  |  | -1+2 | Man | 9.02 | 0.28 | 0.927 |
|  |  |  |  |  |  | Woman | 9.05 | 0.27 |  |

**Experiment 3 Supplemental Tables and Analyses**

**Table S10 Analysis of variance (ANOVA) results on CoDG width in Experiment 3 without age included as a covariate.**

|  | Source | F | df | Sig. | *η_p_^2^* |
| --- | --- | --- | --- | --- | --- |
| Trait-neutral | Desirability (D) | 0.584 | 1, 347 | 0.445 | 0.002 |
|  | Participant gender (G) | 2.224 | 1, 347 | 0.137 | 0.006 |
|  | Face sex (S) | 3.009 | 1, 347 | 0.084 | 0.009 |
|  | D × G | 1.332 | 1, 347 | 0.249 | 0.004 |
|  | D × S | 3.835 | 1, 347 | 0.051 | 0.011 |
|  | G × S | 1.270 | 1, 347 | 0.260 | 0.004 |
|  | D × G × S | 0.093 | 1, 347 | 0.761 | 0.000 |
| Trait-manipulated | Desirability (D) | 1.598 | 1, 347 | 0.207 | 0.005 |
|  | Participant gender (G) | 0.824 | 1, 347 | 0.365 | 0.002 |
|  | Face sex (S) | 0.529 | 1, 347 | 0.468 | 0.002 |
|  | D × G | 1.005 | 1, 347 | 0.317 | 0.003 |
|  | D × S | 5.380* | 1, 347 | 0.021 | 0.015 |
|  | G × S | 2.342 | 1, 347 | 0.127 | 0.007 |
|  | D × G × S | 0.000 | 1, 347 | 0.993 | 0.000 |

**Table S11** **Analysis of variance (ANOVA) results on ratings of trait judgment and likeability in Experiment 3.**

| Task | | Source | F | df | Sig. | *η_p_^2^* |
| --- | --- | --- | --- | --- | --- | --- |
| Trait judgment check | Words implying desirable traits | Desirability (D) | 6846.01*** | 1, 347 | < 0.001 | 0.952 |
|  |  | Participant gender (G) | 0.797 | 1, 347 | 0.373 | 0.002 |
|  |  | Face sex (S) | 0.254 | 1, 347 | 0.615 | 0.001 |
|  |  | D × G | 2.774 | 1, 347 | 0.097 | 0.008 |
|  |  | D × S | 4.65* | 1, 347 | 0.032 | 0.013 |
|  |  | G × S | 0.177 | 1, 347 | 0.674 | 0.001 |
|  |  | D × G × S | 0.002 | 1, 347 | 0.961 | 0.000 |
|  | Words implying undesirable traits | Desirability (D) | 4452.47*** | 1, 347 | < 0.001 | 0.928 |
|  |  | Participant gender (G) | 0.000 | 1, 347 | 0.986 | 0.000 |
|  |  | Face sex (S) | 1.386 | 1, 347 | 0.240 | 0.004 |
|  |  | D × G | 5.35* | 1, 347 | 0.021 | 0.015 |
|  |  | D × S | 0.029 | 1, 347 | 0.865 | 0.000 |
|  |  | G × S | 0.023 | 1, 347 | 0.879 | 0.000 |
|  |  | D × G × S | 0.040 | 1, 347 | 0.842 | 0.000 |
| Likeability rating | Trait-neutral | Desirability (D) | 0.033 | 1, 346 | 0.855 | 0.000 |
|  |  | Participant gender (G) | 0.796 | 1, 346 | 0.373 | 0.002 |
|  |  | Face sex (S) | 19.26*** | 1, 346 | < 0.001 | 0.053 |
|  |  | D × G | 2.404 | 1, 346 | 0.122 | 0.007 |
|  |  | D × S | 0.050 | 1, 346 | 0.823 | 0.000 |
|  |  | G × S | 0.120 | 1, 346 | 0.730 | 0.000 |
|  |  | D × G × S | 0.001 | 1, 346 | 0.975 | 0.000 |
|  | Trait-manipulated | Desirability (D) | 247.80*** | 1, 346 | < 0.001 | 0.417 |
|  |  | Participant gender (G) | 0.341 | 1, 346 | 0.560 | 0.001 |
|  |  | Face sex (S) | 3.583 | 1, 346 | 0.059 | 0.010 |
|  |  | D × G | 1.799 | 1, 346 | 0.181 | 0.005 |
|  |  | D × S | 5.15* | 1, 346 | 0.024 | 0.015 |
|  |  | G × S | 0.667 | 1, 346 | 0.415 | 0.002 |
|  |  | D × G × S | 0.244 | 1, 346 | 0.622 | 0.001 |

**Supplemental Analysis 3.** **Discussion of the trait judgment check in Experiment 3.**

For words implying desirable traits, a significant main effect of desirability [*F*_(1,347)_ = 6846.01, *p* < 0.001, *η_p_*^2^ = 0.95] showed that these words were rated as better describing the likeable people [*M* = 33.40, *SE* = 0.22] than the unlikeable people [*M* = 6.70, *SE* = 0.24]. Additionally, there was a significant interaction between desirability and face sex [*F*_(1,347)_ = 4.65, *p* = 0.032, *η_p_*^2^ = 0.01]. The effect of desirability was significant for both female faces and male faces [*p*s < 0.001], but it was greater for female faces [*M*_+_ = 33.83, *SE* = 0.31; *M*_−_ = 6.43, *SE* = 0.32] than male faces [*M*_+_ = 32.97, *SE* = 0.31; *M*_−_ = 6.96, *SE* = 0.35]. Further analyses showed that the words implying desirable traits were rated as marginally better describing likeable female faces than likeable male faces [*p* = 0.051].

For words implying undesirable traits, a significant main effect of desirability [*F*_(1,347)_ = 4452.47, *p* < 0.001, *η_p_*^2^ = 0.93] showed that these words were rated as better describing the unlikeable people [*M* = 29.42, *SE* = 0.26] than the likeable people [*M* = 6.06, *SE* = 0.24]. Additionally, there was a significant interaction between desirability and participant gender [*F*_(1,347)_ = 5.35, *p* = 0.021, *η_p_*^2^ = 0.02]. The effect of desirability was significant for both men and women [*p*s < 0.001], but it was greater for women [*M*_+_ = 5.66, *SE* = 0.34; *M*_−_ = 29.83, *SE* = 0.36] than men [*M*_+_ = 6.46, *SE* = 0.34; *M*_−_ = 29.02, *SE* = 0.37]. However, the breakdown of the interaction did not reveal statistically significant differences between women and men in their ratings regarding unlikeable faces [*p* = 0.113] or likeable faces [*p* = 0.093].

**Table S12** **Results of paired-samples T tests for the block order effect in Experiment 3.**

| Pair | Block order | Mean | SE | t | df | Sig. | d' |
| --- | --- | --- | --- | --- | --- | --- | --- |
| Neutral-desirable | Block1 | 9.10 | 0.21 | 5.65 | 189 | < 0.001 | 0.41 |
|  | Block2 | 8.33 | 0.20 |  |  |  |  |
| Neutral-undesirable | Block1 | 8.84 | 0.20 | 7.02 | 164 | < 0.001 | 0.55 |
|  | Block2 | 7.95 | 0.20 |  |  |  |  |
|  |  |  |  |  |  |  |  |

Supplemental Results and Discussion. In Experiment 2, we observed that participants’ CoDG width was wider for the identity in the first block compared to the identity in the second block, indicating a training effect. Thus, we wanted to check whether this effect existed also in Experiment 3 by conducting two paired-samples T tests with block order (block 1: trait-neutral vs. block 2: trait-desirable; block 1: trait-neutral vs. block 2: trait-undesirable). For the group of participants who viewed a trait-neutral identity (block 1) followed by a desirable identity (block 2), paired-samples T test showed that CoDG width was wider in block 1 [*M* = 9.10, *SE* = 0.21] than in block 2 [*M* = 8.33, *SE* = 0.20; *t*(189) = 5.65, *p* < 0.001, Cohen’s *d* = 0.41]. Also for the group of participants who viewed a trait-neutral identity (block 1) followed by a undesirable identity (block 2), paired-samples T test showed that CoDG width was wider in block 1 [*M* = 8.84, *SE* = 0.20] than in block 2 [*M* = 7.95, *SE* = 0.20; *t*(164) = 7.02, *p* < 0.001, Cohen’s *d* = 0.55].

In Discussion of Experiment 2, it was noted that CoDG width could narrow quickly due to a training effect. Consistent with this, Experiment 3 observed a training effect on CoDG width, with a wider gaze cone for the face presented in the first block compared to the face presented in the second block. This effect was seen on participants who viewed a trait-neutral identity followed by a likeable identity as well as on participants who viewed a trait-neutral identity followed by an unlikeable identity. However, this finding contrasts with a previous study involving over 400 gaze direction judgment trials, which reported that participants became more liberal in their judgments, resulting in gaze cone widening over time (Collova et al., 2017). It appears that either a training effect or a fatigue effect may occur in eye contact judgment tasks, depending on the number of experimental trials. Specifically, if the task is short, as in the present study, a training effect may emerge. Conversely, a fatigue effect might be observed in longer tasks, such as in Collova et al.’s study (2017). Therefore, a study design that manipulates experimental conditions across different blocks could confound CoDG width with either a training effect or a fatigue effect. Consequently, findings based on comparisons of CoDG width in such designs should be interpreted with caution.

References

Britz, S., Rader, L., Gauggel, S., & Mainz, V. (2022). An English list of trait words including valence, social desirability, and observability ratings. *Behavior Research Methods*, *55*(5), 2669–2686. https://doi.org/10.3758/s13428-022-01921-5

Collova, J. R., Kloth, N., Crookes, K., Burton, N., Chan, C. Y., Hsiao, J. H., & Rhodes, G. (2017). A new other-race effect for gaze perception. *Journal of Experimental Psychology: Human Perception and Performance*, *43*(11), 1857. http://dx.doi.org/10.1037/xhp0000460
